# What you see is what you get: webcam placement influences perception and social coordination

**DOI:** 10.3389/fpsyg.2015.00306

**Published:** 2015-03-19

**Authors:** Laura E. Thomas, Daniel Pemstein

**Affiliations:** ^1^Center for Visual and Cognitive Neuroscience, Department of Psychology, North Dakota State UniversityFargo, ND, USA; ^2^Department of Criminal Justice and Political Science, North Dakota State UniversityFargo, ND, USA

**Keywords:** height perception, power, computer-mediated communication, social coordination

## Abstract

Building on a well-established link between elevation and social power, we demonstrate that—when perceptual information is limited—subtle visual cues can shape people’s representations of others and, in turn, alter strategic social behavior. A cue to elevation (unrelated to physical size) provided by the placement of web cameras in a video chat biased individuals’ perceptions of a partner’s height (Experiment 1) and shaped the extent to which they made decisions in their own self-interest: participants tended to coordinate their behavior in a manner that benefitted the preferences of a partner pictured from a low camera angle during a game of asymmetric coordination (Experiment 2). Our results suggest that people are vulnerable to the influence of a limited viewpoint when forming representations of others in a manner that shapes their strategic choices.

## Introduction

Imagine you have an important job interview that your prospective employers wish to conduct over a video chat program. You check that the audio and video are working on your webcam, clear the visible clutter from your desk, and ensure that the room’s lighting is flattering. But where should you place the camera? The default position at the top of your monitor seems natural, but is it optimal? While video chat allows your interviewers to see and hear you in real time, it limits their ability to sample information outside of the range captured by your static camera and microphone, potentially introducing perceptual cues about you that are not necessarily grounded in physical reality. Indeed, given the perceptual limitations of the interface, these cues may cause viewers to invalidly assign physical characteristics to the person on the other end of the line. Here, we show that the availability of perceptual cues can shape people’s representations of others and in turn bias their social interactions.

To explore this phenomenon, we leverage a well-documented association between elevation and power. People who are physically taller tend to enjoy greater status than their shorter counterparts, serving in more high-ranking occupations (Egolf and Corder, 1991; Melamed and Bozionelos, 1992) and earning larger salaries (Frieze et al., 1990). Words associated with power are more quickly identified when they appear higher in space than lower in space (Schubert, 2005) and people who are depicted as elevated appear more powerful (e.g., Schwartz et al., 1982; Meier et al., 2007). In addition, powerful people overestimate their own height (Duguid and Goncalo, 2012), while people primed to think of their own power perceive others as being shorter (Yap et al., 2013). This association carries over into the vertical angle of photographs: the media portrays powerful individuals from low camera angles (Giessner et al., 2011) and pictures of people taken from below eye-height level tend to be rated as stronger and more active than pictures of the same people taken from above eye-height (Kraft, 1987).

Although a wealth of evidence points to links between real or perceived elevation and social power, few researchers have investigated the extent to which these associations impact actual social decision making behavior. A recent study suggests that associations between elevation and power might shape not only social evaluations, but also interactions: observers asked to act as real estate agents in a lab setting tended to place higher social status clients into higher elevation housing options (Tower-Richardi et al., 2014). However, most research has examined the elevation-power association with self-reported evaluations of height or power, or indirect reaction time measures (e.g., Schubert, 2005; Schubert et al., 2013). We ask if implicit associations between elevation and power are strong enough to alter decision-making in social situations when choices have real consequences for actors, such as when money is on the line. Furthermore, while the majority of examinations of elevation-power associations do not clearly establish the spatial relationship between an observer’s egocentric location and a target’s spatial setup—participants typically evaluate an image on a computer screen abstracted from their own spatial context—recent work points to the need to explore power dynamics in encounters that more clearly approximate real-world spatial mappings (Schubert et al., 2013): looking up to a life-sized picture of a person does not uniformly activate the typical association between elevation and power. Here, we examine how perceptual cues about actors’ relative spatial relationships influence social behavior.

We extend existing research on associations between elevation and the conceptualization of power, using webcam placement to generate perceptual cues to height that situate observers in an illusory spatial context. We demonstrate that alterations in webcam placement create perceptual height illusions (Experiment 1). We then show evidence that providing these illusory perceptual cues to elevation biases observers’ high-level strategic decision-making to favor perceptually taller individuals when playing a classic asymmetrical social coordination game with real-world monetary stakes (Experiment 2). These results further specify how the nature of perceived spatial relationships between people can provide cues that influence personally relevant social decisions.

## Experiment 1

To establish the potential for alterations in camera placement to create an illusion of elevation, we asked participants in Experiment 1 to view images of the same individuals depicted from two different angles—one in which a webcam was placed below the face and another in which a webcam was placed above the face—and then estimate the height of the pictured person.

### Method

**FIGURE 1:**
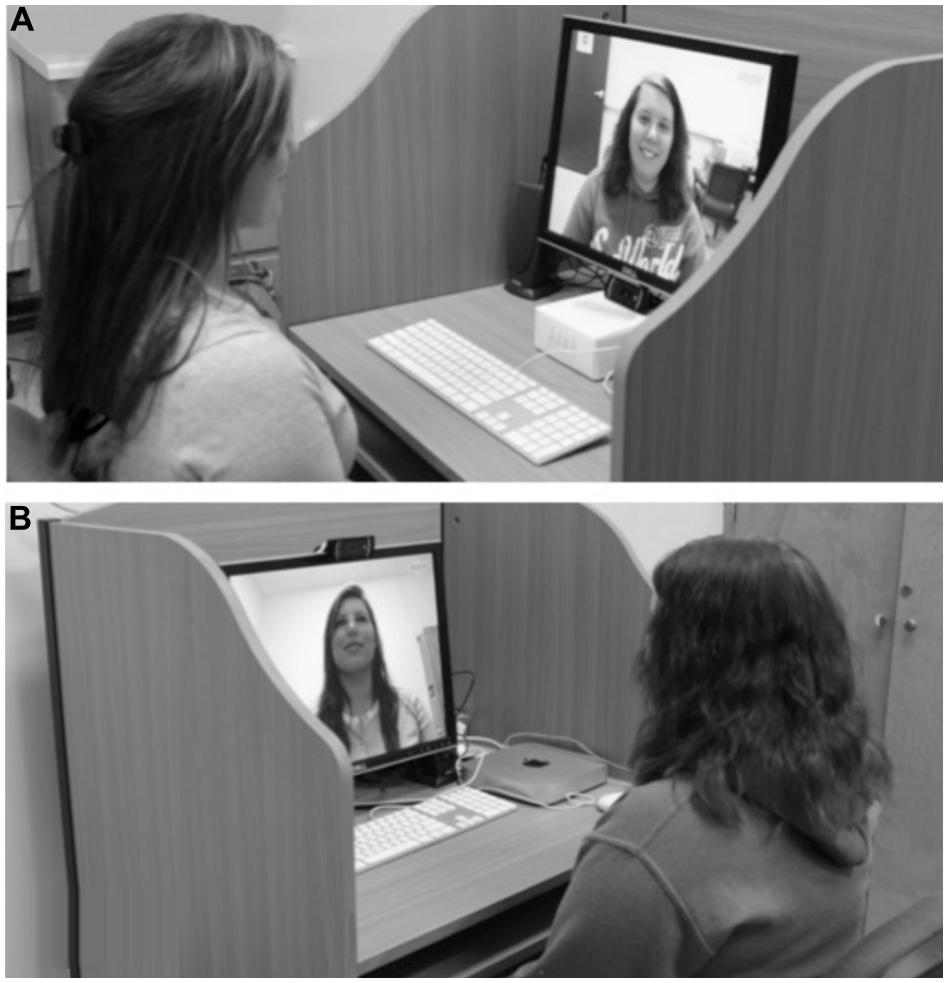
Webcam views for Experiments 1 and 2. **(A)** The face on the monitor is captured from the high camera angle. **(B)** The face on the monitor is captured from the low camera angle.

Eighty-four undergraduate volunteers from North Dakota State University between the ages of 18 and 27 viewed a screen capture image of a face with a neutral expression on a computer monitor. Sample size was set based on the availability of participants during a fixed time period during a semester at NDSU. Participants looked at the face for as long as they wished before providing a verbal estimate of how tall they thought the person in the picture was. The same two faces—one male, one female—served as stimuli, although each participant viewed only a single image. Each face was captured from two web camera positions using the Skype video chat program. In the low camera condition, the camera sat 17 cm below the center of the monitor and was angled up to capture the face image, while in the high camera condition, the camera sat 17 cm above the center of the monitor and was angled down (see **Figure 1**). The physical location of the face remained constant across images—that is, the face occupied the same position on the monitor that participants viewed—yet the angle from which participants viewed these images meant that those participants viewing a low camera image experienced a perceptual cue of looking *up* at the face, while those viewing a high camera image experienced a perceptual cue of looking *down* at the face. An equal number of participants viewed each of the four face images (i.e., 21 participants viewed the male low camera face, 21 participants viewed the male high camera face, 21 participants viewed the female low camera face, and 21 participants viewed the male low camera face); presentation of the faces was randomized across participants.

### Results and Discussion

To account for real differences between the pictured male and female’s heights, for each height estimate, we calculated the ratio of the signed error to the pictured person’s actual height. Participants estimated that people shown in the low camera condition (mean height = 175.79 cm, SD = 8.18 cm) were significantly taller (mean error = +3.1 cm) than the same people shown in the high camera condition (mean height = 171.93 cm, SD = 8.99 cm; mean error = -0.8 cm), *t*(82) = -2.44, *p* = 0.017, *d* = -.53 [-0.99, -0.12]. These results suggest that differences in webcam placement can create disparities in the perceived height of a pictured individual: the same person appears taller when viewed from a low angle than when viewed from a higher angle.

While film theorists have long suspected that camera angles shape the way audiences feel about characters (e.g., Arnheim, 1957), here we show that the vagaries of webcam placement can also have an impact on perceived physical characteristics. The camera is essentially the eye of an audience, and although viewers are aware they are looking at a captured image, they are nonetheless apparently swayed to interpret this image as if it came directly from their own eyes. When the camera looked up at another person—providing a fixed and limited view—our participants perceived this person as taller, presumably experiencing the camera angle as a proxy for their own eye-height that created a cue to elevation.

## Experiment 2

**FIGURE 2:**
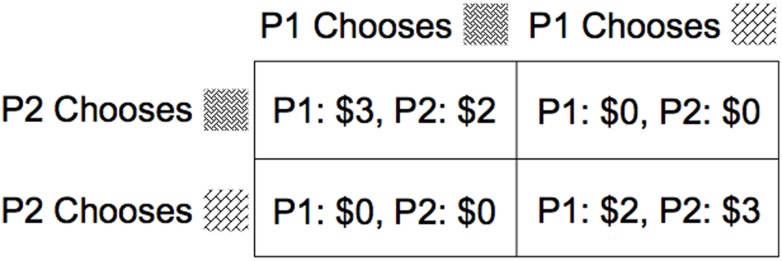
Payoff structure of the coordination game in Experiment 2.

The limited perceptual information provided by the fixed window of a webcam can alter how observers represent the physical characteristics of a pictured person. In Experiment 2, we investigated whether these differences in camera placement would also influence social decision-making. Participants played an asymmetrical social coordination game with the payoff structure depicted in **Figure 2**. In this two-player game, each participant may choose one of two options. As can be seen in the figure, if both players choose the same option, each receives a reward—with the player whose payoff-maximizing pattern is selected receiving $3 to the other player’s $2—while if they choose different patterns, neither receives a payoff. The players are motivated to coordinate with one another—they only receive a monetary reward under circumstances in which they choose the same option—yet each option for working together presents an outcome that favors one player over the other. Most game theorists refer to this game as “Battle of the Sexes.” We examined whether the manipulations in webcam placement that influenced height perception in Experiment 1 would bias participants toward coordinating around options that were most beneficial to the player depicted in the low camera condition—that is, the player whose partner looked up at her.

### Method

Eighty-four pairs of undergraduate volunteers between the ages of 18 and 26 from North Dakota State University participated for course credit and were randomly assigned to either an asymmetrical or symmetrical camera condition. Sample size was set to match the size in Experiment 1 (i.e., 84 pairs in Experiment 2 versus 84 individuals in Experiment 1). Participants were seated in separate rooms—never seeing each other face-to-face—in front of identical computer monitors attached to speakers and a web camera. Forty-two of these pairs experienced an asymmetry in camera placement across rooms identical to the manipulations of Experiment 1: one player was captured from a low camera angle while the other was captured from a high camera angle (see **Figure 1**). As can be seen in the figure, in this asymmetrical condition, the player captured from the low camera angle experienced perceptual information about her partner implying a spatial relationship in which she looked *down* at the other person, while the player captured from the high camera angle instead had a perceptual experience of looking *up* to the other person. The remaining 42 pairs of volunteers participated in a symmetrical control condition in which the cameras in both rooms sat in the standard position at the top of the monitor (i.e., both players were in a high camera condition). An experimenter connected the two computers in a Skype video chat session before participants arrived and then turned off the monitors and muted the speakers. After a participant was seated, the experimenter turned the participant away from the monitor and helped her adjust the height of the chair so that top of the participant’s head was aligned with the top of the computer monitor. If needed, the experimenter also adjusted the tilt of the webcam to center the image of the participant’s head on the screen, keeping the physical location of each participant’s image the same across displays. These procedures helped ensure that perceptual information related to real differences in participants’ heights was minimized in our presentation. In the asymmetrical condition, the players experienced opposing perceptual cues to elevation, creating a situation in which the implied spatial relationship between participants was of one player sitting *above* the other, while in the symmetrical condition, players experienced identical cues.

The experimenter gave each participant an instruction sheet describing the coordination game, its payoff scheme, and two geometric patterns. After completing the call, each player would choose either the pattern that maximized his or her own payoff or the pattern that maximized the other player’s payoff. Pairs of sheets were arranged so that each participant’s payoff-maximizing pattern appeared on the left side of the page without a verbal label. We wished to avoid creating a possible focal point based upon pattern labels or locations that could lead participants to coordinate around one pattern more frequently than another (Schelling, 1960). Our results indicated that participants were not biased in their selection of one pattern over another, *χ*^2^(1) = 0.15, *p* = 0.700, *w* = 0.03 [0.02,0.14]. Payoff-maximizing pattern was counterbalanced across conditions.

After both participants read the instructions and reported understanding the rules of the game, the experimenter turned the speakers on, stood outside of the testing rooms, instructed participants to face and look into the monitors for their chat, and started a timer. Participants chatted freely for 2 min—discussing a coordination strategy—before the experimenter disconnected the call and provided each participant with a pen to make their pattern choices on the instruction sheet. After they made their responses, participants completed a post-test questionnaire that asked them what they thought the study was investigating, what the purpose of the study was, and what they predicted the results of the study would be. They then received payment based on the outcome of the coordination game and were debriefed.

### Results and Discussion

**Table 1** summarizes the choices all participants made in the coordination game. We coded each participant’s response as a dichotomous variable—ones for payoff-maximizing ($3) and zeros for non-payoff-maximizing ($2) choices. Note that this breakdown includes games in which players failed to coordinate, leading to choices in **Table 1** displayed between groups that do not total 100%. We used logistic regression to examine the relationship between this dependent variable and factors of condition (asymmetrical/symmetrical) and room (A/B—participants in the asymmetrical condition were captured by the low camera in Room A and the high camera in Room B, while the camera was high for both participants in the symmetrical condition) as well as the condition by room interaction. The results of this regression, depicted in **Table 2**, show that, while room assignment seems to have shaped choices in the asymmetric condition, room did not have a substantial influence on behavior in the symmetric condition. Interpreting **Table 2**, the coefficient capturing the effect of room (i.e., low camera versus high camera) for participants in the asymmetric condition—is negative and statistically significant, indicating that participants who experienced the implied spatial relationship of looking *up* to the other player chose the payoff-maximizing option less often than their counterparts who experienced perceptual cues consistent with looking *down* on the other player. Furthermore, the coefficient for the interaction between condition and room is positive and statistically significant, showing that room assignment had less influence on participants’ choices in the symmetric condition than the asymmetric condition. An examination of **Table 1** shows—and the logistic regression validates—that while room assignment substantially influenced behavior in the asymmetric condition, it had essentially no impact upon participants’ choices in the symmetric condition.

**Table 1:** Participant choice in Experiment 2.

Condition	Room	Percentage Choosing $3 Option
Asymmetric webcam	A (low camera; *n* = 21)	67%
	B (high camera; *n* = 21)	38%
Symmetric webcam	A (high camera; *n* = 21)	50%
	B (high camera; *n* = 21)	52%


**Table 2:** Results of logistic regression of participant choice on condition and room.

	Coefficient	SE	*P*-value	95% CI
Intercept	0.69	0.33	0.034	(0.05, 1.33)
Room	-1.18	0.46	0.010	(-2.07, -0.28)
Condition	-0.69	0.45	0.123	(-1.57, 0.19)
Room × Condition	1.27	0.63	0.044	(0.04, 2.51)

A weakness of this individual-level logistic regression model is that it assumes that individuals’ responses are independent of each other, while in the coordination game, responses within pairs are clearly correlated. At the individual level, this is not an easy issue to address using standard tools; for instance, including a random intercept for negotiating pair would not appropriately model the tendency of coordinating pairs to make diverging choices. Therefore, we also analyzed the data treating pairs as the level of analysis, and conducted a test to see if the proportion of coordinating pairs in which the Room A participant obtained the higher payoff differed across the symmetric (24 pairs) and asymmetric (17 pairs) conditions (χ^2^ = 2.38, df = 1, *p* = 0.061, 95% CI = (-0.01, 1.00), Cohen’s h = 0.37 [-0.09, 0.84]). This marginal result, from a less powerful analysis, which dropped six non-coordinating pairs in the asymmetric condition and seven in the symmetric condition, provides converging evidence for our argument.

No participants in the asymmetric condition wrote about the disparity in webcam placement in the post-test questionnaire, suggesting they were not overtly aware of this perceptual manipulation or its impact on their behavior.

These results suggest that participants captured by the high camera chose the pattern that would give themselves the smaller payoff almost twice as often as participants captured by the low camera in the asymmetric condition. In other words, participants who experienced perceptual cues consistent with a spatial relationship in which their partners were above them more frequently deferred to the choice that would potentially benefit their partner more than themselves, yet participants who instead saw cues suggesting their partners were below them tended to make choices that reflected their own self-interests. Importantly, our symmetric control condition suggests that this effect was specific to camera placement and not a product of room assignment; any differences inherent in the testing rooms remained constant across conditions. Because only participants in the asymmetric high-camera condition experienced a visual cue about their partner that differed from other participants, the overall effect was likely driven by their deference. While we did not directly assess height in this experiment due to the potential for reports about height to color choices in the decision-making game (or vice versa), the fact that we found no effect of room in the control condition suggests that a height illusion may lie behind the influence of webcam placement in the asymmetric condition. Although the cues to elevation provided by webcam placement were unrelated to participants’ actual size or the physical location of their images on a display, these subtle perceptual cues seem to have triggered power associations that substantially influenced higher-level decision-making.

## General Discussion

The results of our experiments suggest that when perceptual information is limited—as in video chat—differences in camera placement can alter observers’ representations of other people in ways that color the strategic social decisions they make. In Experiment 1, participants who viewed an image of a face captured by a low-placed camera perceived the pictured person as being taller than participants who instead saw the same face from a higher-placed camera. In Experiment 2, we found that these cues to elevation, cues completely unrelated to physical size, substantially biased the extent to which people made decisions in their own self-interest in a video chat. More specifically, participants who had a perceptual experience of looking up at their partners tended to act against their own interests more often than those that looked down at the other person.

Our results add to the growing literature on associations between elevation and power, showing that, regardless of actual elevation, cues associated with a *perceived* difference in elevation between participants in an interpersonal interaction may substantially tip the scales of a bargaining outcome. Previous research has demonstrated ties between schematized representations of elevation and power judgments (e.g., Schubert, 2005) and suggested that people may be evolutionary predisposed to interpret physical height as indicating power (Fiske, 2004). Semantic and episodic primes to power can induce people to behave in a more goal-directed manner (for a review, see Smith and Galinsky, 2010) and take the initial steps in negotiations (Magee et al., 2007). We have shown that a perceptual cue to elevation—one that influenced height judgments and presumably created an impression of domination—can likewise serve as a prime that influences behavior, at least in cases in which perceptual information is limited, as is the case in a video chat. Although participants’ perception of their partner’s height was not assessed—a potential limitation of the current research—our results across experiments nonetheless demonstrate that webcam placement has an influence on both height perception and social decision making.

Interestingly, when individuals have a firm sense of the power dynamics in an encounter, social interactions tend to run more smoothly (e.g., Smith and Galinsky, 2010). Participants in Experiment 2 had a limited time to coordinate their behavior with a stranger, so they may have unconsciously used the cues available to them to quickly establish a power dynamic to facilitate their interaction. Participants could not make direct eye contact—they could look into the camera or fixate their partner’s face, but were unable to simultaneously do both—preventing either player from engaging in visual dominance behavior (e.g., Dovidio and Ellyson, 1985; Linkey and Firestone, 1990). The limited head-and-shoulders view the webcams provided likewise gave participants little information about gesture or body posture that could have served as cues to power (Hall et al., 2005). In the asymmetric condition, camera placement was perhaps one of the strongest cues participants could instantly access to establish a power hierarchy before they even began discussing the game, giving players captured by the low camera the upper hand in negotiations. When this cue was unavailable in the symmetric condition participants instead may have relied more on the non-verbal and verbal cues we did not manipulate in order to delegate a power structure, leading to the more equal distribution of choices between rooms.

The results of the current study have interesting implications for the broader literature on associations between elevation and power. While recent work examining observers’ self-reported levels of respect for targets suggests that looking up to a life-sized picture of a person does not uniformly activate the typical association between elevation and power when additional cues to the target’s achievement are available (Schubert et al., 2013), our results suggest that artificial perceptual cues to elevation are sufficient to trigger these associations in a face-to-face encounter via webcam. It is important to note that we found a significant influence of camera placement on behavior even though participants in our study were able to briefly converse with each other, potentially picking up on other cues to power such as wealth or social standing, pointing to the strength of our perceptual manipulation to influence behavior in the face of other sources of variance. The fact that this manipulation influenced behavior on a social task in which participants held real-world personal stakes compliments recent work demonstrating the strength of elevation-power associations to guide daily decisions (Tower-Richardi et al., 2014), even outside of conscious awareness.

Our results also have implications for the literature on game theoretic models of social coordination. While “cheap talk” communication has long been known to reduce problems of equilibrium selection in asymmetric coordination games like the one our participants played (e.g., Dawes et al., 1977; Cooper et al., 1989), our data also suggest that people are driven to coordinate around outcomes that are grounded in unconscious perceptual factors.

Participants’ rates of coordination were uniformly high, consistent with the idea that face-to-face (and webcam-to-webcam) contact built a sense of rapport that facilitated coordination (Drolet and Morris, 2000). However, a sense of rapport or responsibility (e.g., Sonsino and Sirota, 2003) cannot explain why participants specifically chose to coordinate around an option that favored the player captured by the low camera. In symmetric coordination games in which the equilibrium payoffs are identical across players, introducing a focal point that is salient to all players aids coordination (Schelling, 1960), but in asymmetric games players often ignore focal points in hopes of securing their most preferred outcome (Crawford et al., 2008). In our study, however, despite asymmetry in the game, participants were able to coordinate around the focal point of camera angle, overwhelmingly behaving in a way that favored the player that was looked up to. Thus, we not only provide some of the first evidence that subtle perceptual cues can guide equilibrium selection, but also demonstrate the efficacy of such cues in a class of games where players are known to ignore obvious focal points—games that characterize a multitude of social interactions ranging from routine face-to-face negotiations to decision making by actors in large financial networks. Furthermore, while our study examined a situation in which outright coordination failure is unlikely, our results imply that people can reduce the likelihood of coordination failure in asymmetric games by introducing psychologically resonant focal points, suggesting a potentially fruitful avenue for future research that has largely been overlooked in experimental studies of coordination failure (for a review, see Devetag and Ortmann, 2007).

In addition to demonstrating the power of relatively subtle perceptual cues to shape social decision-making, our findings also have applied practical relevance. The technological advances making videoconferencing widely available have introduced a new mode of commonplace human interaction. Video chat mimics many aspects of face-to-face conversation, increasing feelings of connectedness between people communicating remotely (Ames et al., 2010; Kirk et al., 2010; Neustaedter and Greenberg, 2011), but these interactions often fail to live up to the experience of in-person encounters (Bos et al., 2002; Nguyen and Canny, 2007, 2009). As issues of cost, convenience, and environmental concerns drive increasing numbers of people to employ this imperfect alternative to face-to-face conversation, it is important to understand the ways in which this mode of communication shapes interactions. The conditions under which people view a chat partner shape their representations of this partner—fooling perception into seeing physical characteristics that are not necessarily there. Our study suggests that the next time you are planning an important video chat call, you may be able to use camera placement to your distinct advantage.

## Acknowledgments and Replication Information

North Dakota EPSCoR and NSF Grant EPS-0814442 and NIGMS #P20 GM103505 provided financial support for this research. We thank Nicole Hartle, Christopher Held, Alexa Johnson, Nathan Ochsner, Marissa Weinkauf, and Sylvia Ziejewski for their assistance with data collection. The replication package for this study can be found at: http://thedata.harvard.edu/dvn/faces/study/StudyPage.xhtml?globalId=doi:10.7910/DVN/29310.

## Conflict of Interest Statement

The authors declare that the research was conducted in the absence of any commercial or financial relationships that could be construed as a potential conflict of interest.

## References

[B1] Ames M. G. Go J. Kaye J. Spasojevic M. (2010). “Making love in the network closet: the benefits and work of family videochat,”in *Proceedings of the 2010 ACM Conference on Computer Supported Cooperative Work*, New York, NY.

[B2] Arnheim R. (1957). *Film as Art*. Berkeley, CA: University of California Press.

[B3] Bos N. Olson J. Gergle D. Olson G. Wright Z. (2002). “Effects of four computer-mediated communications channels on trust development,” in *Proceedings of the SIGCHI conference on Human Factors in Computing Systems*, New York, NY.

[B4] Cooper R. DeJong D. V. Forsythe R. Ross T. W. (1989). Communication in the battle of the sexes game: some experimental results. *RAND J. Econ.* 20 568–587 10.2307/2555734

[B5] Crawford V. P. Gneezy U. Rottenstreich Y. (2008). The power of focal points is limited: even minute payoff asymmetry may yield large coordination failures. *Am. Econ. Rev.* 98 1443–1458 10.1257/aer.98.4.1443

[B6] Dawes R. M. McTavish J. Shaklee H. (1977). Behavior, communication, and assumptions about other people’s behavior in a commons dilemma situation. *J. Pers. Soc. Psychol.* 35 1–11 10.1037/0022-3514.35.1.1

[B7] Devetag G. Ortmann A. (2007). When and why? A critical survey on coordination failure in the laboratory. *Exp. Econ.* 10 331–344 10.1007/s10683-007-9178-9

[B8] Dovidio J. F. Ellyson S. L. (1985). “Patterns of visual dominance behavior in humans,” in *Power, Dominance, and Nonverbal Behavior,* eds Ellyson S. L. Dovidio J. F. (New York, NY: Springer-Verlag), 129–149 10.1007/978-1-4612-5106-4_7

[B9] Drolet A. L. Morris M. W. (2000). Rapport in conflict resolution: accounting for how face-to-face contact fosters mutual cooperation in mixed-motive conflicts. *J. Exp. Soc. Psychol.* 36 26–50 10.1006/jesp.1999.1395

[B10] Duguid M. M. Goncalo J. A. (2012). Living large: the powerful overestimate their own height. *Psychol. Sci.* 23 36–40 10.1177/0956797611422915 22173738

[B11] Egolf D. B. Corder L. E. (1991). Height differences of low and high job status, female and male corporate employees. *Sex Roles* 24 365–373 10.1007/BF00288309

[B12] Fiske A. P. (2004). “Four modes of constituting relationships: consubstantial assimilation; space, magnitude, time and force; concrete procedures; abstract symbolism,” in *Relational Models Theory: A Contemporary Overview*, ed. Haslam N. (Mahwah, NJ: Erlbaum), 61–146.

[B13] Frieze I. H. Olson J. E. Good D. C. (1990). Perceived and actual discrimination in the salaries of male and female managers. *J. Appl. Soc. Psychol.* 20 46–67 10.1111/j.1559-1816.1990.tb00377.x

[B14] Giessner S. R. Ryan M. K. Schubert T. W. (2011). The power of pictures: vertical picture angles in power pictures. *Media Psychol.* 14 441–463 10.1080/15213269.2011.620541

[B15] Hall J. A. LeBeau L. S. Coats E. J. (2005). Nonverbal behavior and the vertical dimension of social relations: a meta-analysis. *Psychol. Bull.* 131 898–924 10.1037/0033-2909.131.6.898 16351328

[B16] Kirk D. S. Sellen A. Cao X. (2010). “Home video communication: mediating closeless,” In *Proceedings of the 2010 ACM conference on computer supported cooperative work* (New York, NY: Association for Computing Machinery, Inc.).

[B17] Kraft R. N. (1987). The influence of camera angle on comprehension and retention of pictorial events. *Mem. Cogn.* 15 291–307 10.3758/BF03197032 3670051

[B18] Linkey H. E. Firestone J. J. (1990). Dyad dominance composition effects, nonverbal behaviors, and influence. *J. Res. Pers.* 24 206–215 10.1016/0092-6566(90)90017-Z

[B19] Magee J. C. Galinsky A. D. Gruenfeld D. H. (2007). Power, propensity to negotiate, and moving first in competitive interactions. *Personal. Soc. Psychol. Bull.* 33 200–212 10.1177/0146167206294413 17259581

[B20] Meier B. P. Hauser D. J. Robinson M. D. Frisen C. K. Schjeldahl K. (2007). What’s “up” with god? Vertical space as a representation of the divine. *J. Pers. Soc. Psychol.* 93 699–710 10.1037/0022-3514.93.5.699 17983295

[B21] Melamed T. Bozionelos N. (1992). Managerial promotion and height. *Psychol. Rep.* 71 979–986 10.2466/pr0.1992.71.3.979 1454952

[B22] Neustaedter C. Greenberg S.(2011). “Intimacy in long-distance relationships over video chat,” in *Proceedings of the SIGCHI Conference on Human Factors in Computing Systems*, New York, NY.

[B23] Nguyen D. T. Canny J. (2007). “Multiview: improving trust in group video conferencing through spatial faithfulness,” in *Proceedings of the SIGCHI Conference on Human Factors in Computing Systems*, New York, NY. 10.1145/1240624.1240846

[B24] Nguyen D. T. Canny J. (2009). “More than face-to-face: empathy effects of video framing,” in *Proceedings of the SIGCHI Conference on Human Factors in Computing Systems*, New York NY.

[B25] Schelling T. (1960). *The Strategy of Conflict*. Cambridge, MA: Harvard University Press.

[B26] Schubert L. Schubert T. W. Topolinski S. (2013). The effect of spatial elevation on respect depends on merit and medium. *Soc. Psychol.* 44 147–159 10.1027/1864-9335/a000134

[B27] Schubert T. W. (2005). Your highness: vertical positions as perceptual symbols of power. *J. Pers. Soc. Psychol.* 89 1–21 10.1037/0022-3514.89.1.1 16060739

[B28] Schwartz B. Tesser A. Powell E. (1982). Dominance cues in nonverbal behavior. *Soc. Psychol. Q.* 45 114–120 10.2307/3033934

[B29] Smith P. K. Galinsky A. D. (2010). The nonconscious nature of power: cues and consequences. *Soc. Personal. Psychol. Compass* 4 918–938 10.1111/j.1751-9004.2010.00300.x

[B30] Sonsino D. Sirota J. (2003). Strategic pattern recognition—experimental evidence. *Games Econ. Behav.* 44 390–411 10.1016/S0899-8256(03)00040-X

[B31] Tower-Richardi S. M. Brunye T. T. Gagnon S. A. Mahoney C. R. Taylor H. A. (2014). Living the high life: social status influences real estate decision making. *J. Appl. Soc. Psychol.* 44 611–621 10.1111/jasp.12253

[B32] Yap A. J. Mason M. F. Ames D. R. (2013). The powerful size others down: the link between power and estimates of others’ size. *J. Exp. Soc. Psychol.* 49 591–594 10.1016/j.jesp.2012.10.003

